# Supplementary material for: Pyruvate Dehydrogenase Kinase 1 inhibition mediated oxidative phosphorylation enhancement in cartilage promotes osteoarthritis progression
Source: BMC Musculoskelet Disord. 2023 Jul 20;24:597. doi: 10.1186/s12891-023-06585-6 (PMC10357736; doi:10.1186/s12891-023-06585-6)
Supplement: Supplementary file 1 — Additional file 1. [file 12891_2023_6585_MOESM1_ESM.zip › BP.Gsea.1653623667859/GOBP_RIBONUCLEOPROTEIN_COMPLEX_BIOGENESIS.html]

Details for gene set GOBP\_RIBONUCLEOPROTEIN\_COMPLEX\_BIOGENESIS[GSEA]

|  || Dataset | 55457\_collapsed\_to\_symbols.cls.cls #OA\_versus\_normal.cls.cls #OA\_versus\_normal\_repos |
| Phenotype | cls.cls#OA\_versus\_normal\_repos |
| Upregulated in class | normal |
| GeneSet | GOBP\_RIBONUCLEOPROTEIN\_COMPLEX\_BIOGENESIS |
| Enrichment Score (ES) | -0.3589367 |
| Normalized Enrichment Score (NES) | -1.8191893 |
| Nominal p-value | 0.0 |
| FDR q-value | 0.08618452 |
| FWER p-Value | 0.959 |
Table: GSEA Results Summary

  

Fig 1: Enrichment plot: GOBP\_RIBONUCLEOPROTEIN\_COMPLEX\_BIOGENESIS      
 Profile of the Running ES Score & Positions of GeneSet Members on the Rank Ordered List

  

| SYMBOL | TITLE | RANK IN GENE LIST | RANK METRIC SCORE | RUNNING ES | CORE ENRICHMENT || 1 | NVL | nuclear VCP like [Source:HGNC Symbol;Acc:HGNC:8070] | 73 | 0.956 | 0.0020 | No |
| 2 | LSM4 | "LSM4 homolog, U6 small nuclear RNA and mRNA degradation associated [Source:HGNC Symbol;Acc:HGNC:17259]" | 174 | 0.837 | 0.0008 | No |
| 3 | BUD23 | BUD23 rRNA methyltransferase and ribosome maturation factor [Source:HGNC Symbol;Acc:HGNC:16405] | 210 | 0.813 | 0.0046 | No |
| 4 | NMD3 | NMD3 ribosome export adaptor [Source:HGNC Symbol;Acc:HGNC:24250] | 240 | 0.791 | 0.0088 | No |
| 5 | RPP30 | ribonuclease P/MRP subunit p30 [Source:HGNC Symbol;Acc:HGNC:17688] | 265 | 0.777 | 0.0132 | No |
| 6 | TXNL4A | thioredoxin like 4A [Source:HGNC Symbol;Acc:HGNC:30551] | 321 | 0.736 | 0.0148 | No |
| 7 | SRPK3 | SRSF protein kinase 3 [Source:HGNC Symbol;Acc:HGNC:11402] | 329 | 0.734 | 0.0202 | No |
| 8 | IMP3 | IMP U3 small nucleolar ribonucleoprotein 3 [Source:HGNC Symbol;Acc:HGNC:14497] | 381 | 0.714 | 0.0220 | No |
| 9 | POP5 | "POP5 homolog, ribonuclease P/MRP subunit [Source:HGNC Symbol;Acc:HGNC:17689]" | 494 | 0.662 | 0.0185 | No |
| 10 | MCTS1 | MCTS1 re-initiation and release factor [Source:HGNC Symbol;Acc:HGNC:23357] | 527 | 0.649 | 0.0212 | No |
| 11 | METTL18 | methyltransferase like 18 [Source:HGNC Symbol;Acc:HGNC:28793] | 685 | 0.597 | 0.0135 | No |
| 12 | NGRN | "neugrin, neurite outgrowth associated [Source:HGNC Symbol;Acc:HGNC:18077]" | 741 | 0.583 | 0.0139 | No |
| 13 | CLP1 | cleavage factor polyribonucleotide kinase subunit 1 [Source:HGNC Symbol;Acc:HGNC:16999] | 748 | 0.582 | 0.0182 | No |
| 14 | CRNKL1 | crooked neck pre-mRNA splicing factor 1 [Source:HGNC Symbol;Acc:HGNC:15762] | 803 | 0.568 | 0.0185 | No |
| 15 | PRPF18 | pre-mRNA processing factor 18 [Source:HGNC Symbol;Acc:HGNC:17351] | 836 | 0.559 | 0.0205 | No |
| 16 | NPM3 | nucleophosmin/nucleoplasmin 3 [Source:HGNC Symbol;Acc:HGNC:7931] | 868 | 0.551 | 0.0225 | No |
| 17 | SRSF9 | serine and arginine rich splicing factor 9 [Source:HGNC Symbol;Acc:HGNC:10791] | 882 | 0.548 | 0.0259 | No |
| 18 | DDX23 | DEAD-box helicase 23 [Source:HGNC Symbol;Acc:HGNC:17347] | 913 | 0.538 | 0.0279 | No |
| 19 | USP39 | ubiquitin specific peptidase 39 [Source:HGNC Symbol;Acc:HGNC:20071] | 991 | 0.519 | 0.0260 | No |
| 20 | DHX29 | DExH-box helicase 29 [Source:HGNC Symbol;Acc:HGNC:15815] | 1012 | 0.515 | 0.0286 | No |
| 21 | POP4 | "POP4 homolog, ribonuclease P/MRP subunit [Source:HGNC Symbol;Acc:HGNC:30081]" | 1015 | 0.514 | 0.0327 | No |
| 22 | XRCC5 | X-ray repair cross complementing 5 [Source:HGNC Symbol;Acc:HGNC:12833] | 1018 | 0.513 | 0.0367 | No |
| 23 | GTF2H5 | general transcription factor IIH subunit 5 [Source:HGNC Symbol;Acc:HGNC:21157] | 1032 | 0.511 | 0.0398 | No |
| 24 | SNRPD3 | small nuclear ribonucleoprotein D3 polypeptide [Source:HGNC Symbol;Acc:HGNC:11160] | 1046 | 0.508 | 0.0429 | No |
| 25 | RPF1 | ribosome production factor 1 homolog [Source:HGNC Symbol;Acc:HGNC:30350] | 1061 | 0.504 | 0.0459 | No |
| 26 | CD2BP2 | CD2 cytoplasmic tail binding protein 2 [Source:HGNC Symbol;Acc:HGNC:1656] | 1158 | 0.485 | 0.0422 | No |
| 27 | EIF2D | eukaryotic translation initiation factor 2D [Source:HGNC Symbol;Acc:HGNC:6583] | 1162 | 0.485 | 0.0459 | No |
| 28 | PRKRA | protein activator of interferon induced protein kinase EIF2AK2 [Source:HGNC Symbol;Acc:HGNC:9438] | 1222 | 0.472 | 0.0451 | No |
| 29 | SNRPD2 | small nuclear ribonucleoprotein D2 polypeptide [Source:HGNC Symbol;Acc:HGNC:11159] | 1290 | 0.460 | 0.0435 | No |
| 30 | MRPS11 | mitochondrial ribosomal protein S11 [Source:HGNC Symbol;Acc:HGNC:14050] | 1305 | 0.457 | 0.0461 | No |
| 31 | MRPL22 | mitochondrial ribosomal protein L22 [Source:HGNC Symbol;Acc:HGNC:14480] | 1311 | 0.456 | 0.0494 | No |
| 32 | GEMIN2 | gem nuclear organelle associated protein 2 [Source:HGNC Symbol;Acc:HGNC:10884] | 1322 | 0.454 | 0.0523 | No |
| 33 | POP7 | "POP7 homolog, ribonuclease P/MRP subunit [Source:HGNC Symbol;Acc:HGNC:19949]" | 1337 | 0.452 | 0.0549 | No |
| 34 | TRMT112 | tRNA methyltransferase activator subunit 11-2 [Source:HGNC Symbol;Acc:HGNC:26940] | 1359 | 0.449 | 0.0568 | No |
| 35 | PSIP1 | PC4 and SFRS1 interacting protein 1 [Source:HGNC Symbol;Acc:HGNC:9527] | 1482 | 0.431 | 0.0506 | No |
| 36 | NSA2 | NSA2 ribosome biogenesis factor [Source:HGNC Symbol;Acc:HGNC:30728] | 1508 | 0.428 | 0.0521 | No |
| 37 | ZNHIT3 | zinc finger HIT-type containing 3 [Source:HGNC Symbol;Acc:HGNC:12309] | 1648 | 0.407 | 0.0444 | No |
| 38 | MRPS7 | mitochondrial ribosomal protein S7 [Source:HGNC Symbol;Acc:HGNC:14499] | 1650 | 0.406 | 0.0476 | No |
| 39 | CLNS1A | chloride nucleotide-sensitive channel 1A [Source:HGNC Symbol;Acc:HGNC:2080] | 1671 | 0.403 | 0.0493 | No |
| 40 | RRP8 | ribosomal RNA processing 8 [Source:HGNC Symbol;Acc:HGNC:29030] | 1673 | 0.402 | 0.0525 | No |
| 41 | EMG1 | EMG1 N1-specific pseudouridine methyltransferase [Source:HGNC Symbol;Acc:HGNC:16912] | 1688 | 0.401 | 0.0546 | No |
| 42 | NUDT21 | nudix hydrolase 21 [Source:HGNC Symbol;Acc:HGNC:13870] | 1734 | 0.393 | 0.0542 | No |
| 43 | YJU2 | YJU2 splicing factor homolog [Source:HGNC Symbol;Acc:HGNC:25518] | 1832 | 0.380 | 0.0496 | No |
| 44 | C1D | C1D nuclear receptor corepressor [Source:HGNC Symbol;Acc:HGNC:29911] | 1848 | 0.379 | 0.0515 | No |
| 45 | MRM2 | mitochondrial rRNA methyltransferase 2 [Source:HGNC Symbol;Acc:HGNC:16352] | 1880 | 0.375 | 0.0521 | No |
| 46 | EXOSC7 | exosome component 7 [Source:HGNC Symbol;Acc:HGNC:28112] | 1969 | 0.364 | 0.0480 | No |
| 47 | NHP2 | NHP2 ribonucleoprotein [Source:HGNC Symbol;Acc:HGNC:14377] | 2026 | 0.358 | 0.0465 | No |
| 48 | DROSHA | drosha ribonuclease III [Source:HGNC Symbol;Acc:HGNC:17904] | 2054 | 0.356 | 0.0472 | No |
| 49 | NOP10 | NOP10 ribonucleoprotein [Source:HGNC Symbol;Acc:HGNC:14378] | 2068 | 0.354 | 0.0491 | No |
| 50 | METTL5 | "methyltransferase 5, N6-adenosine [Source:HGNC Symbol;Acc:HGNC:25006]" | 2107 | 0.349 | 0.0489 | No |
| 51 | MTREX | Mtr4 exosome RNA helicase [Source:HGNC Symbol;Acc:HGNC:18734] | 2138 | 0.346 | 0.0493 | No |
| 52 | GTF3A | general transcription factor IIIA [Source:HGNC Symbol;Acc:HGNC:4662] | 2151 | 0.344 | 0.0512 | No |
| 53 | TBL3 | transducin beta like 3 [Source:HGNC Symbol;Acc:HGNC:11587] | 2154 | 0.344 | 0.0538 | No |
| 54 | TARBP2 | TARBP2 subunit of RISC loading complex [Source:HGNC Symbol;Acc:HGNC:11569] | 2311 | 0.325 | 0.0440 | No |
| 55 | UTP14C | UTP14C small subunit processome component [Source:HGNC Symbol;Acc:HGNC:20321] | 2336 | 0.322 | 0.0447 | No |
| 56 | XPO1 | exportin 1 [Source:HGNC Symbol;Acc:HGNC:12825] | 2477 | 0.305 | 0.0361 | No |
| 57 | EFL1 | elongation factor like GTPase 1 [Source:HGNC Symbol;Acc:HGNC:25789] | 2501 | 0.303 | 0.0367 | No |
| 58 | PIH1D1 | PIH1 domain containing 1 [Source:HGNC Symbol;Acc:HGNC:26075] | 2667 | 0.283 | 0.0259 | No |
| 59 | RUVBL2 | RuvB like AAA ATPase 2 [Source:HGNC Symbol;Acc:HGNC:10475] | 2778 | 0.272 | 0.0193 | No |
| 60 | TSR2 | TSR2 ribosome maturation factor [Source:HGNC Symbol;Acc:HGNC:25455] | 2924 | 0.258 | 0.0099 | No |
| 61 | RPUSD2 | RNA pseudouridine synthase domain containing 2 [Source:HGNC Symbol;Acc:HGNC:24180] | 2934 | 0.257 | 0.0112 | No |
| 62 | REXO4 | "REX4 homolog, 3'-5' exonuclease [Source:HGNC Symbol;Acc:HGNC:12820]" | 2953 | 0.255 | 0.0119 | No |
| 63 | C1QBP | complement C1q binding protein [Source:HGNC Symbol;Acc:HGNC:1243] | 3083 | 0.241 | 0.0036 | No |
| 64 | SF3A1 | splicing factor 3a subunit 1 [Source:HGNC Symbol;Acc:HGNC:10765] | 3152 | 0.235 | 0.0001 | No |
| 65 | GEMIN8 | gem nuclear organelle associated protein 8 [Source:HGNC Symbol;Acc:HGNC:26044] | 3156 | 0.235 | 0.0017 | No |
| 66 | PUF60 | poly(U) binding splicing factor 60 [Source:HGNC Symbol;Acc:HGNC:17042] | 3237 | 0.227 | -0.0028 | No |
| 67 | PRMT7 | protein arginine methyltransferase 7 [Source:HGNC Symbol;Acc:HGNC:25557] | 3250 | 0.226 | -0.0019 | No |
| 68 | FRG1 | FSHD region gene 1 [Source:HGNC Symbol;Acc:HGNC:3954] | 3254 | 0.226 | -0.0003 | No |
| 69 | DDX1 | DEAD-box helicase 1 [Source:HGNC Symbol;Acc:HGNC:2734] | 3305 | 0.221 | -0.0025 | No |
| 70 | AGO1 | argonaute RISC component 1 [Source:HGNC Symbol;Acc:HGNC:3262] | 3430 | 0.208 | -0.0107 | No |
| 71 | SNRPF | small nuclear ribonucleoprotein polypeptide F [Source:HGNC Symbol;Acc:HGNC:11162] | 3509 | 0.201 | -0.0152 | No |
| 72 | ERCC2 | "ERCC excision repair 2, TFIIH core complex helicase subunit [Source:HGNC Symbol;Acc:HGNC:3434]" | 3544 | 0.198 | -0.0163 | No |
| 73 | NSUN3 | NOP2/Sun RNA methyltransferase 3 [Source:HGNC Symbol;Acc:HGNC:26208] | 3612 | 0.192 | -0.0201 | No |
| 74 | RIOK2 | RIO kinase 2 [Source:HGNC Symbol;Acc:HGNC:18999] | 3615 | 0.192 | -0.0187 | No |
| 75 | SNRPC | small nuclear ribonucleoprotein polypeptide C [Source:HGNC Symbol;Acc:HGNC:11157] | 3649 | 0.190 | -0.0198 | No |
| 76 | COIL | coilin [Source:HGNC Symbol;Acc:HGNC:2184] | 3684 | 0.187 | -0.0210 | No |
| 77 | GEMIN6 | gem nuclear organelle associated protein 6 [Source:HGNC Symbol;Acc:HGNC:20044] | 3741 | 0.182 | -0.0240 | No |
| 78 | ZRSR2 | "zinc finger CCCH-type, RNA binding motif and serine/arginine rich 2 [Source:HGNC Symbol;Acc:HGNC:23019]" | 3771 | 0.179 | -0.0248 | No |
| 79 | CUL4B | cullin 4B [Source:HGNC Symbol;Acc:HGNC:2555] | 3783 | 0.179 | -0.0242 | No |
| 80 | SNU13 | small nuclear ribonucleoprotein 13 [Source:HGNC Symbol;Acc:HGNC:7819] | 3795 | 0.178 | -0.0237 | No |
| 81 | RPS27L | ribosomal protein S27 like [Source:HGNC Symbol;Acc:HGNC:18476] | 3882 | 0.166 | -0.0292 | No |
| 82 | LSM6 | "LSM6 homolog, U6 small nuclear RNA and mRNA degradation associated [Source:HGNC Symbol;Acc:HGNC:17017]" | 4019 | 0.154 | -0.0387 | No |
| 83 | GTPBP10 | GTP binding protein 10 [Source:HGNC Symbol;Acc:HGNC:25106] | 4129 | 0.143 | -0.0462 | No |
| 84 | DIS3 | "DIS3 homolog, exosome endoribonuclease and 3'-5' exoribonuclease [Source:HGNC Symbol;Acc:HGNC:20604]" | 4187 | 0.139 | -0.0497 | No |
| 85 | RIOK3 | RIO kinase 3 [Source:HGNC Symbol;Acc:HGNC:11451] | 4206 | 0.137 | -0.0500 | No |
| 86 | FBL | fibrillarin [Source:HGNC Symbol;Acc:HGNC:3599] | 4219 | 0.135 | -0.0498 | No |
| 87 | SDAD1 | SDA1 domain containing 1 [Source:HGNC Symbol;Acc:HGNC:25537] | 4229 | 0.134 | -0.0495 | No |
| 88 | EXOSC5 | exosome component 5 [Source:HGNC Symbol;Acc:HGNC:24662] | 4295 | 0.128 | -0.0536 | No |
| 89 | AGO4 | argonaute RISC component 4 [Source:HGNC Symbol;Acc:HGNC:18424] | 4346 | 0.124 | -0.0566 | No |
| 90 | PRPF6 | pre-mRNA processing factor 6 [Source:HGNC Symbol;Acc:HGNC:15860] | 4567 | 0.108 | -0.0732 | No |
| 91 | RPL26L1 | ribosomal protein L26 like 1 [Source:HGNC Symbol;Acc:HGNC:17050] | 4584 | 0.106 | -0.0736 | No |
| 92 | DDX28 | DEAD-box helicase 28 [Source:HGNC Symbol;Acc:HGNC:17330] | 4602 | 0.105 | -0.0741 | No |
| 93 | ERI3 | ERI1 exoribonuclease family member 3 [Source:HGNC Symbol;Acc:HGNC:17276] | 4627 | 0.102 | -0.0752 | No |
| 94 | LSM2 | "LSM2 homolog, U6 small nuclear RNA and mRNA degradation associated [Source:HGNC Symbol;Acc:HGNC:13940]" | 4641 | 0.101 | -0.0754 | No |
| 95 | ABT1 | activator of basal transcription 1 [Source:HGNC Symbol;Acc:HGNC:17369] | 4783 | 0.088 | -0.0859 | No |
| 96 | DDX47 | DEAD-box helicase 47 [Source:HGNC Symbol;Acc:HGNC:18682] | 4789 | 0.088 | -0.0856 | No |
| 97 | MRM3 | mitochondrial rRNA methyltransferase 3 [Source:HGNC Symbol;Acc:HGNC:18485] | 4806 | 0.087 | -0.0862 | No |
| 98 | EIF2S2 | eukaryotic translation initiation factor 2 subunit beta [Source:HGNC Symbol;Acc:HGNC:3266] | 4838 | 0.084 | -0.0880 | No |
| 99 | KAT2B | lysine acetyltransferase 2B [Source:HGNC Symbol;Acc:HGNC:8638] | 4867 | 0.082 | -0.0895 | No |
| 100 | ZCCHC4 | zinc finger CCHC-type containing 4 [Source:HGNC Symbol;Acc:HGNC:22917] | 4909 | 0.079 | -0.0922 | No |
| 101 | UTP3 | UTP3 small subunit processome component [Source:HGNC Symbol;Acc:HGNC:24477] | 4934 | 0.077 | -0.0934 | No |
| 102 | RPL10A | ribosomal protein L10a [Source:HGNC Symbol;Acc:HGNC:10299] | 4974 | 0.074 | -0.0959 | No |
| 103 | MPHOSPH6 | M-phase phosphoprotein 6 [Source:HGNC Symbol;Acc:HGNC:7214] | 5077 | 0.067 | -0.1035 | No |
| 104 | EIF4H | eukaryotic translation initiation factor 4H [Source:HGNC Symbol;Acc:HGNC:12741] | 5118 | 0.062 | -0.1062 | No |
| 105 | PRPF8 | pre-mRNA processing factor 8 [Source:HGNC Symbol;Acc:HGNC:17340] | 5287 | 0.049 | -0.1192 | No |
| 106 | STRAP | serine/threonine kinase receptor associated protein [Source:HGNC Symbol;Acc:HGNC:30796] | 5375 | 0.042 | -0.1258 | No |
| 107 | NSUN5 | NOP2/Sun RNA methyltransferase 5 [Source:HGNC Symbol;Acc:HGNC:16385] | 5434 | 0.038 | -0.1301 | No |
| 108 | UTP18 | UTP18 small subunit processome component [Source:HGNC Symbol;Acc:HGNC:24274] | 5522 | 0.031 | -0.1368 | No |
| 109 | SNRPE | small nuclear ribonucleoprotein polypeptide E [Source:HGNC Symbol;Acc:HGNC:11161] | 5551 | 0.029 | -0.1388 | No |
| 110 | NOPCHAP1 | NOP protein chaperone 1 [Source:HGNC Symbol;Acc:HGNC:28628] | 5552 | 0.029 | -0.1385 | No |
| 111 | PIN4 | "peptidylprolyl cis/trans isomerase, NIMA-interacting 4 [Source:HGNC Symbol;Acc:HGNC:8992]" | 5567 | 0.027 | -0.1394 | No |
| 112 | CDC73 | cell division cycle 73 [Source:HGNC Symbol;Acc:HGNC:16783] | 5604 | 0.023 | -0.1421 | No |
| 113 | METTL17 | methyltransferase like 17 [Source:HGNC Symbol;Acc:HGNC:19280] | 5673 | 0.018 | -0.1474 | No |
| 114 | SART1 | "spliceosome associated factor 1, recruiter of U4/U6.U5 tri-snRNP [Source:HGNC Symbol;Acc:HGNC:10538]" | 5690 | 0.017 | -0.1485 | No |
| 115 | EXOSC4 | exosome component 4 [Source:HGNC Symbol;Acc:HGNC:18189] | 5693 | 0.016 | -0.1486 | No |
| 116 | TFB1M | "transcription factor B1, mitochondrial [Source:HGNC Symbol;Acc:HGNC:17037]" | 5789 | 0.009 | -0.1561 | No |
| 117 | RPS19 | ribosomal protein S19 [Source:HGNC Symbol;Acc:HGNC:10402] | 5952 | -0.002 | -0.1689 | No |
| 118 | NUP88 | nucleoporin 88 [Source:HGNC Symbol;Acc:HGNC:8067] | 5976 | -0.003 | -0.1708 | No |
| 119 | NOL11 | nucleolar protein 11 [Source:HGNC Symbol;Acc:HGNC:24557] | 5984 | -0.003 | -0.1713 | No |
| 120 | MRPL20 | mitochondrial ribosomal protein L20 [Source:HGNC Symbol;Acc:HGNC:14478] | 5989 | -0.004 | -0.1716 | No |
| 121 | RSL24D1 | ribosomal L24 domain containing 1 [Source:HGNC Symbol;Acc:HGNC:18479] | 5992 | -0.004 | -0.1717 | No |
| 122 | SF3A2 | splicing factor 3a subunit 2 [Source:HGNC Symbol;Acc:HGNC:10766] | 5999 | -0.004 | -0.1722 | No |
| 123 | SUV39H1 | suppressor of variegation 3-9 homolog 1 [Source:HGNC Symbol;Acc:HGNC:11479] | 6028 | -0.007 | -0.1743 | No |
| 124 | MPHOSPH10 | M-phase phosphoprotein 10 [Source:HGNC Symbol;Acc:HGNC:7213] | 6088 | -0.011 | -0.1789 | No |
| 125 | EIF6 | eukaryotic translation initiation factor 6 [Source:HGNC Symbol;Acc:HGNC:6159] | 6140 | -0.015 | -0.1829 | No |
| 126 | ERAL1 | Era like 12S mitochondrial rRNA chaperone 1 [Source:HGNC Symbol;Acc:HGNC:3424] | 6158 | -0.017 | -0.1841 | No |
| 127 | ATM | ATM serine/threonine kinase [Source:HGNC Symbol;Acc:HGNC:795] | 6179 | -0.019 | -0.1855 | No |
| 128 | ISG20 | interferon stimulated exonuclease gene 20 [Source:HGNC Symbol;Acc:HGNC:6130] | 6212 | -0.022 | -0.1879 | No |
| 129 | TRMT61B | tRNA methyltransferase 61B [Source:HGNC Symbol;Acc:HGNC:26070] | 6223 | -0.022 | -0.1885 | No |
| 130 | TAF9 | TATA-box binding protein associated factor 9 [Source:HGNC Symbol;Acc:HGNC:11542] | 6297 | -0.027 | -0.1941 | No |
| 131 | NOP53 | NOP53 ribosome biogenesis factor [Source:HGNC Symbol;Acc:HGNC:4333] | 6334 | -0.031 | -0.1967 | No |
| 132 | NGDN | neuroguidin [Source:HGNC Symbol;Acc:HGNC:20271] | 6370 | -0.033 | -0.1993 | No |
| 133 | RPL35A | ribosomal protein L35a [Source:HGNC Symbol;Acc:HGNC:10345] | 6383 | -0.034 | -0.1999 | No |
| 134 | AAR2 | AAR2 splicing factor [Source:HGNC Symbol;Acc:HGNC:15886] | 6401 | -0.035 | -0.2010 | No |
| 135 | RBFA | ribosome binding factor A [Source:HGNC Symbol;Acc:HGNC:26120] | 6473 | -0.040 | -0.2063 | No |
| 136 | RPL10L | ribosomal protein L10 like [Source:HGNC Symbol;Acc:HGNC:17976] | 6502 | -0.042 | -0.2082 | No |
| 137 | DENR | density regulated re-initiation and release factor [Source:HGNC Symbol;Acc:HGNC:2769] | 6611 | -0.050 | -0.2164 | No |
| 138 | CUL4A | cullin 4A [Source:HGNC Symbol;Acc:HGNC:2554] | 6701 | -0.055 | -0.2231 | No |
| 139 | NOL8 | nucleolar protein 8 [Source:HGNC Symbol;Acc:HGNC:23387] | 6709 | -0.056 | -0.2232 | No |
| 140 | METTL25B | methyltransferase like 25B [Source:HGNC Symbol;Acc:HGNC:24273] | 6774 | -0.061 | -0.2278 | No |
| 141 | RPP38 | ribonuclease P/MRP subunit p38 [Source:HGNC Symbol;Acc:HGNC:30329] | 6814 | -0.064 | -0.2303 | No |
| 142 | EIF2S3 | eukaryotic translation initiation factor 2 subunit gamma [Source:HGNC Symbol;Acc:HGNC:3267] | 6870 | -0.069 | -0.2342 | No |
| 143 | EIF5 | eukaryotic translation initiation factor 5 [Source:HGNC Symbol;Acc:HGNC:3299] | 6934 | -0.074 | -0.2386 | No |
| 144 | MTERF4 | mitochondrial transcription termination factor 4 [Source:HGNC Symbol;Acc:HGNC:28785] | 6972 | -0.078 | -0.2409 | No |
| 145 | FCF1 | FCF1 rRNA-processing protein [Source:HGNC Symbol;Acc:HGNC:20220] | 6999 | -0.080 | -0.2423 | No |
| 146 | UTP11 | UTP11 small subunit processome component [Source:HGNC Symbol;Acc:HGNC:24329] | 7015 | -0.081 | -0.2429 | No |
| 147 | TFB2M | "transcription factor B2, mitochondrial [Source:HGNC Symbol;Acc:HGNC:18559]" | 7112 | -0.089 | -0.2498 | No |
| 148 | SNRNP200 | small nuclear ribonucleoprotein U5 subunit 200 [Source:HGNC Symbol;Acc:HGNC:30859] | 7249 | -0.099 | -0.2598 | No |
| 149 | NLE1 | notchless homolog 1 [Source:HGNC Symbol;Acc:HGNC:19889] | 7253 | -0.099 | -0.2592 | No |
| 150 | WBP11 | WW domain binding protein 11 [Source:HGNC Symbol;Acc:HGNC:16461] | 7312 | -0.103 | -0.2630 | No |
| 151 | DCAF13 | DDB1 and CUL4 associated factor 13 [Source:HGNC Symbol;Acc:HGNC:24535] | 7459 | -0.115 | -0.2737 | No |
| 152 | RUVBL1 | RuvB like AAA ATPase 1 [Source:HGNC Symbol;Acc:HGNC:10474] | 7477 | -0.116 | -0.2741 | No |
| 153 | NOL3 | nucleolar protein 3 [Source:HGNC Symbol;Acc:HGNC:7869] | 7545 | -0.122 | -0.2785 | No |
| 154 | RPL35 | ribosomal protein L35 [Source:HGNC Symbol;Acc:HGNC:10344] | 7563 | -0.124 | -0.2788 | No |
| 155 | IMP4 | IMP U3 small nucleolar ribonucleoprotein 4 [Source:HGNC Symbol;Acc:HGNC:30856] | 7643 | -0.130 | -0.2840 | No |
| 156 | EIF4A3 | eukaryotic translation initiation factor 4A3 [Source:HGNC Symbol;Acc:HGNC:18683] | 7646 | -0.130 | -0.2831 | No |
| 157 | TSR3 | TSR3 ribosome maturation factor [Source:HGNC Symbol;Acc:HGNC:14175] | 7676 | -0.133 | -0.2844 | No |
| 158 | GNL3L | G protein nucleolar 3 like [Source:HGNC Symbol;Acc:HGNC:25553] | 7722 | -0.135 | -0.2869 | No |
| 159 | POLR2D | RNA polymerase II subunit D [Source:HGNC Symbol;Acc:HGNC:9191] | 7726 | -0.136 | -0.2860 | No |
| 160 | FASTKD2 | FAST kinase domains 2 [Source:HGNC Symbol;Acc:HGNC:29160] | 7730 | -0.136 | -0.2851 | No |
| 161 | TGS1 | trimethylguanosine synthase 1 [Source:HGNC Symbol;Acc:HGNC:17843] | 7791 | -0.141 | -0.2888 | No |
| 162 | MRPS2 | mitochondrial ribosomal protein S2 [Source:HGNC Symbol;Acc:HGNC:14495] | 7863 | -0.146 | -0.2932 | No |
| 163 | NOL10 | nucleolar protein 10 [Source:HGNC Symbol;Acc:HGNC:25862] | 7880 | -0.146 | -0.2933 | No |
| 164 | CPSF6 | cleavage and polyadenylation specific factor 6 [Source:HGNC Symbol;Acc:HGNC:13871] | 7885 | -0.147 | -0.2924 | No |
| 165 | PRPF31 | pre-mRNA processing factor 31 [Source:HGNC Symbol;Acc:HGNC:15446] | 7898 | -0.147 | -0.2922 | No |
| 166 | PRMT5 | protein arginine methyltransferase 5 [Source:HGNC Symbol;Acc:HGNC:10894] | 7904 | -0.148 | -0.2914 | No |
| 167 | DDX49 | DEAD-box helicase 49 [Source:HGNC Symbol;Acc:HGNC:18684] | 7945 | -0.151 | -0.2933 | No |
| 168 | DDX18 | DEAD-box helicase 18 [Source:HGNC Symbol;Acc:HGNC:2741] | 7996 | -0.154 | -0.2961 | No |
| 169 | EIF4B | eukaryotic translation initiation factor 4B [Source:HGNC Symbol;Acc:HGNC:3285] | 8051 | -0.159 | -0.2991 | No |
| 170 | NOL6 | nucleolar protein 6 [Source:HGNC Symbol;Acc:HGNC:19910] | 8081 | -0.161 | -0.3001 | No |
| 171 | RPL3L | ribosomal protein L3 like [Source:HGNC Symbol;Acc:HGNC:10351] | 8097 | -0.163 | -0.2999 | No |
| 172 | MAK16 | MAK16 homolog [Source:HGNC Symbol;Acc:HGNC:13703] | 8098 | -0.163 | -0.2986 | No |
| 173 | DHX30 | DExH-box helicase 30 [Source:HGNC Symbol;Acc:HGNC:16716] | 8111 | -0.164 | -0.2982 | No |
| 174 | KRR1 | KRR1 small subunit processome component homolog [Source:HGNC Symbol;Acc:HGNC:5176] | 8126 | -0.165 | -0.2980 | No |
| 175 | MTERF3 | mitochondrial transcription termination factor 3 [Source:HGNC Symbol;Acc:HGNC:24258] | 8144 | -0.166 | -0.2980 | No |
| 176 | NOC4L | nucleolar complex associated 4 homolog [Source:HGNC Symbol;Acc:HGNC:28461] | 8225 | -0.173 | -0.3030 | No |
| 177 | RSL1D1 | ribosomal L1 domain containing 1 [Source:HGNC Symbol;Acc:HGNC:24534] | 8286 | -0.177 | -0.3063 | No |
| 178 | RPP40 | ribonuclease P/MRP subunit p40 [Source:HGNC Symbol;Acc:HGNC:20992] | 8318 | -0.179 | -0.3073 | No |
| 179 | RPS21 | ribosomal protein S21 [Source:HGNC Symbol;Acc:HGNC:10409] | 8394 | -0.185 | -0.3118 | No |
| 180 | SETX | senataxin [Source:HGNC Symbol;Acc:HGNC:445] | 8398 | -0.185 | -0.3105 | No |
| 181 | EXOSC10 | exosome component 10 [Source:HGNC Symbol;Acc:HGNC:9138] | 8404 | -0.186 | -0.3094 | No |
| 182 | WDR12 | WD repeat domain 12 [Source:HGNC Symbol;Acc:HGNC:14098] | 8505 | -0.194 | -0.3158 | No |
| 183 | EXOSC8 | exosome component 8 [Source:HGNC Symbol;Acc:HGNC:17035] | 8554 | -0.198 | -0.3180 | No |
| 184 | UTP6 | UTP6 small subunit processome component [Source:HGNC Symbol;Acc:HGNC:18279] | 8638 | -0.203 | -0.3230 | No |
| 185 | UTP25 | UTP25 small subunit processor component [Source:HGNC Symbol;Acc:HGNC:28440] | 8699 | -0.209 | -0.3260 | No |
| 186 | PWP1 | "PWP1 homolog, endonuclein [Source:HGNC Symbol;Acc:HGNC:17015]" | 8724 | -0.211 | -0.3262 | No |
| 187 | SIRT7 | sirtuin 7 [Source:HGNC Symbol;Acc:HGNC:14935] | 8733 | -0.211 | -0.3251 | No |
| 188 | XAB2 | XPA binding protein 2 [Source:HGNC Symbol;Acc:HGNC:14089] | 8778 | -0.215 | -0.3269 | No |
| 189 | PTGES3 | prostaglandin E synthase 3 [Source:HGNC Symbol;Acc:HGNC:16049] | 8802 | -0.217 | -0.3270 | No |
| 190 | RPSA | ribosomal protein SA [Source:HGNC Symbol;Acc:HGNC:6502] | 8866 | -0.222 | -0.3302 | No |
| 191 | RRP1B | ribosomal RNA processing 1B [Source:HGNC Symbol;Acc:HGNC:23818] | 8909 | -0.225 | -0.3317 | No |
| 192 | EBNA1BP2 | EBNA1 binding protein 2 [Source:HGNC Symbol;Acc:HGNC:15531] | 8931 | -0.226 | -0.3315 | No |
| 193 | AATF | apoptosis antagonizing transcription factor [Source:HGNC Symbol;Acc:HGNC:19235] | 8935 | -0.227 | -0.3299 | No |
| 194 | NSUN5P1 | NSUN5 pseudogene 1 [Source:HGNC Symbol;Acc:HGNC:19146] | 8988 | -0.231 | -0.3322 | No |
| 195 | SNRPG | small nuclear ribonucleoprotein polypeptide G [Source:HGNC Symbol;Acc:HGNC:11163] | 9067 | -0.237 | -0.3365 | No |
| 196 | SNRPB | small nuclear ribonucleoprotein polypeptides B and B1 [Source:HGNC Symbol;Acc:HGNC:11153] | 9085 | -0.239 | -0.3359 | No |
| 197 | MRM1 | mitochondrial rRNA methyltransferase 1 [Source:HGNC Symbol;Acc:HGNC:26202] | 9114 | -0.241 | -0.3362 | No |
| 198 | USP4 | ubiquitin specific peptidase 4 [Source:HGNC Symbol;Acc:HGNC:12627] | 9130 | -0.242 | -0.3354 | No |
| 199 | NCBP1 | nuclear cap binding protein subunit 1 [Source:HGNC Symbol;Acc:HGNC:7658] | 9149 | -0.244 | -0.3348 | No |
| 200 | FTSJ3 | FtsJ RNA 2'-O-methyltransferase 3 [Source:HGNC Symbol;Acc:HGNC:17136] | 9193 | -0.247 | -0.3362 | No |
| 201 | ADAR | adenosine deaminase RNA specific [Source:HGNC Symbol;Acc:HGNC:225] | 9195 | -0.247 | -0.3343 | No |
| 202 | BYSL | bystin like [Source:HGNC Symbol;Acc:HGNC:1157] | 9277 | -0.254 | -0.3387 | No |
| 203 | RPS25 | ribosomal protein S25 [Source:HGNC Symbol;Acc:HGNC:10413] | 9282 | -0.254 | -0.3369 | No |
| 204 | HEATR1 | HEAT repeat containing 1 [Source:HGNC Symbol;Acc:HGNC:25517] | 9339 | -0.259 | -0.3393 | No |
| 205 | GEMIN7 | gem nuclear organelle associated protein 7 [Source:HGNC Symbol;Acc:HGNC:20045] | 9441 | -0.268 | -0.3452 | No |
| 206 | GNL2 | G protein nucleolar 2 [Source:HGNC Symbol;Acc:HGNC:29925] | 9457 | -0.269 | -0.3442 | No |
| 207 | RAN | "RAN, member RAS oncogene family [Source:HGNC Symbol;Acc:HGNC:9846]" | 9478 | -0.272 | -0.3435 | No |
| 208 | UTP14A | UTP14A small subunit processome component [Source:HGNC Symbol;Acc:HGNC:10665] | 9536 | -0.277 | -0.3458 | No |
| 209 | SNRPD1 | small nuclear ribonucleoprotein D1 polypeptide [Source:HGNC Symbol;Acc:HGNC:11158] | 9579 | -0.281 | -0.3469 | No |
| 210 | RCL1 | RNA terminal phosphate cyclase like 1 [Source:HGNC Symbol;Acc:HGNC:17687] | 9586 | -0.282 | -0.3451 | No |
| 211 | PRKDC | "protein kinase, DNA-activated, catalytic subunit [Source:HGNC Symbol;Acc:HGNC:9413]" | 9632 | -0.286 | -0.3463 | No |
| 212 | ABCE1 | ATP binding cassette subfamily E member 1 [Source:HGNC Symbol;Acc:HGNC:69] | 9675 | -0.289 | -0.3473 | No |
| 213 | RRP15 | ribosomal RNA processing 15 homolog [Source:HGNC Symbol;Acc:HGNC:24255] | 9775 | -0.299 | -0.3528 | No |
| 214 | WDR74 | WD repeat domain 74 [Source:HGNC Symbol;Acc:HGNC:25529] | 9777 | -0.299 | -0.3504 | No |
| 215 | URB1 | URB1 ribosome biogenesis homolog [Source:HGNC Symbol;Acc:HGNC:17344] | 9833 | -0.304 | -0.3523 | No |
| 216 | GCFC2 | GC-rich sequence DNA-binding factor 2 [Source:HGNC Symbol;Acc:HGNC:1317] | 9908 | -0.312 | -0.3557 | No |
| 217 | NIP7 | nucleolar pre-rRNA processing protein NIP7 [Source:HGNC Symbol;Acc:HGNC:24328] | 9950 | -0.316 | -0.3564 | Yes |
| 218 | RNASEL | ribonuclease L [Source:HGNC Symbol;Acc:HGNC:10050] | 9965 | -0.317 | -0.3549 | Yes |
| 219 | KHDC4 | "KH domain containing 4, pre-mRNA splicing factor [Source:HGNC Symbol;Acc:HGNC:29145]" | 9997 | -0.321 | -0.3547 | Yes |
| 220 | DIMT1 | DIM1 rRNA methyltransferase and ribosome maturation factor [Source:HGNC Symbol;Acc:HGNC:30217] | 10023 | -0.324 | -0.3541 | Yes |
| 221 | SHQ1 | "SHQ1, H/ACA ribonucleoprotein assembly factor [Source:HGNC Symbol;Acc:HGNC:25543]" | 10045 | -0.326 | -0.3531 | Yes |
| 222 | HSP90AB1 | heat shock protein 90 alpha family class B member 1 [Source:HGNC Symbol;Acc:HGNC:5258] | 10060 | -0.327 | -0.3516 | Yes |
| 223 | RRP9 | "ribosomal RNA processing 9, U3 small nucleolar RNA binding protein [Source:HGNC Symbol;Acc:HGNC:16829]" | 10064 | -0.327 | -0.3492 | Yes |
| 224 | GEMIN4 | gem nuclear organelle associated protein 4 [Source:HGNC Symbol;Acc:HGNC:15717] | 10075 | -0.328 | -0.3473 | Yes |
| 225 | DDX51 | DEAD-box helicase 51 [Source:HGNC Symbol;Acc:HGNC:20082] | 10133 | -0.334 | -0.3491 | Yes |
| 226 | RPL5 | ribosomal protein L5 [Source:HGNC Symbol;Acc:HGNC:10360] | 10201 | -0.341 | -0.3517 | Yes |
| 227 | ZNHIT6 | zinc finger HIT-type containing 6 [Source:HGNC Symbol;Acc:HGNC:26089] | 10220 | -0.343 | -0.3503 | Yes |
| 228 | RPL24 | ribosomal protein L24 [Source:HGNC Symbol;Acc:HGNC:10325] | 10263 | -0.347 | -0.3508 | Yes |
| 229 | PES1 | pescadillo ribosomal biogenesis factor 1 [Source:HGNC Symbol;Acc:HGNC:8848] | 10268 | -0.347 | -0.3483 | Yes |
| 230 | SRSF1 | serine and arginine rich splicing factor 1 [Source:HGNC Symbol;Acc:HGNC:10780] | 10272 | -0.348 | -0.3457 | Yes |
| 231 | BRIX1 | biogenesis of ribosomes BRX1 [Source:HGNC Symbol;Acc:HGNC:24170] | 10275 | -0.348 | -0.3430 | Yes |
| 232 | SRSF5 | serine and arginine rich splicing factor 5 [Source:HGNC Symbol;Acc:HGNC:10787] | 10282 | -0.349 | -0.3407 | Yes |
| 233 | MRTO4 | "MRT4 homolog, ribosome maturation factor [Source:HGNC Symbol;Acc:HGNC:18477]" | 10300 | -0.350 | -0.3392 | Yes |
| 234 | SF3A3 | splicing factor 3a subunit 3 [Source:HGNC Symbol;Acc:HGNC:10767] | 10311 | -0.352 | -0.3371 | Yes |
| 235 | LUC7L2 | "LUC7 like 2, pre-mRNA splicing factor [Source:HGNC Symbol;Acc:HGNC:21608]" | 10334 | -0.354 | -0.3360 | Yes |
| 236 | NOL9 | nucleolar protein 9 [Source:HGNC Symbol;Acc:HGNC:26265] | 10361 | -0.357 | -0.3351 | Yes |
| 237 | SCAF11 | SR-related CTD associated factor 11 [Source:HGNC Symbol;Acc:HGNC:10784] | 10375 | -0.358 | -0.3333 | Yes |
| 238 | ISG20L2 | interferon stimulated exonuclease gene 20 like 2 [Source:HGNC Symbol;Acc:HGNC:25745] | 10404 | -0.361 | -0.3325 | Yes |
| 239 | RRP7A | ribosomal RNA processing 7 homolog A [Source:HGNC Symbol;Acc:HGNC:24286] | 10449 | -0.367 | -0.3331 | Yes |
| 240 | SRPK2 | SRSF protein kinase 2 [Source:HGNC Symbol;Acc:HGNC:11306] | 10522 | -0.376 | -0.3357 | Yes |
| 241 | RPS8 | ribosomal protein S8 [Source:HGNC Symbol;Acc:HGNC:10441] | 10525 | -0.376 | -0.3328 | Yes |
| 242 | RPS14 | ribosomal protein S14 [Source:HGNC Symbol;Acc:HGNC:10387] | 10558 | -0.379 | -0.3323 | Yes |
| 243 | DKC1 | dyskerin pseudouridine synthase 1 [Source:HGNC Symbol;Acc:HGNC:2890] | 10591 | -0.382 | -0.3317 | Yes |
| 244 | PA2G4 | proliferation-associated 2G4 [Source:HGNC Symbol;Acc:HGNC:8550] | 10598 | -0.383 | -0.3291 | Yes |
| 245 | EXOSC9 | exosome component 9 [Source:HGNC Symbol;Acc:HGNC:9137] | 10606 | -0.384 | -0.3265 | Yes |
| 246 | RPS7 | ribosomal protein S7 [Source:HGNC Symbol;Acc:HGNC:10440] | 10608 | -0.384 | -0.3235 | Yes |
| 247 | RPP25 | ribonuclease P and MRP subunit p25 [Source:HGNC Symbol;Acc:HGNC:30361] | 10621 | -0.385 | -0.3213 | Yes |
| 248 | DHX9 | DExH-box helicase 9 [Source:HGNC Symbol;Acc:HGNC:2750] | 10627 | -0.386 | -0.3185 | Yes |
| 249 | LUC7L3 | LUC7 like 3 pre-mRNA splicing factor [Source:HGNC Symbol;Acc:HGNC:24309] | 10647 | -0.388 | -0.3169 | Yes |
| 250 | SRSF6 | serine and arginine rich splicing factor 6 [Source:HGNC Symbol;Acc:HGNC:10788] | 10659 | -0.389 | -0.3146 | Yes |
| 251 | RPL10 | ribosomal protein L10 [Source:HGNC Symbol;Acc:HGNC:10298] | 10684 | -0.391 | -0.3133 | Yes |
| 252 | WDR18 | WD repeat domain 18 [Source:HGNC Symbol;Acc:HGNC:17956] | 10685 | -0.391 | -0.3101 | Yes |
| 253 | YTHDC1 | YTH domain containing 1 [Source:HGNC Symbol;Acc:HGNC:30626] | 10687 | -0.391 | -0.3070 | Yes |
| 254 | NOP14 | NOP14 nucleolar protein [Source:HGNC Symbol;Acc:HGNC:16821] | 10747 | -0.398 | -0.3085 | Yes |
| 255 | RPL7A | ribosomal protein L7a [Source:HGNC Symbol;Acc:HGNC:10364] | 10811 | -0.406 | -0.3102 | Yes |
| 256 | CELF2 | CUGBP Elav-like family member 2 [Source:HGNC Symbol;Acc:HGNC:2550] | 10832 | -0.409 | -0.3085 | Yes |
| 257 | RBM5 | RNA binding motif protein 5 [Source:HGNC Symbol;Acc:HGNC:9902] | 10849 | -0.410 | -0.3064 | Yes |
| 258 | SRSF10 | serine and arginine rich splicing factor 10 [Source:HGNC Symbol;Acc:HGNC:16713] | 10926 | -0.422 | -0.3090 | Yes |
| 259 | NOLC1 | nucleolar and coiled-body phosphoprotein 1 [Source:HGNC Symbol;Acc:HGNC:15608] | 10942 | -0.423 | -0.3068 | Yes |
| 260 | SF3B1 | splicing factor 3b subunit 1 [Source:HGNC Symbol;Acc:HGNC:10768] | 10948 | -0.424 | -0.3037 | Yes |
| 261 | NOP16 | NOP16 nucleolar protein [Source:HGNC Symbol;Acc:HGNC:26934] | 10959 | -0.425 | -0.3010 | Yes |
| 262 | RRP12 | ribosomal RNA processing 12 homolog [Source:HGNC Symbol;Acc:HGNC:29100] | 10970 | -0.427 | -0.2984 | Yes |
| 263 | NAT10 | N-acetyltransferase 10 [Source:HGNC Symbol;Acc:HGNC:29830] | 10998 | -0.429 | -0.2970 | Yes |
| 264 | DDX10 | DEAD-box helicase 10 [Source:HGNC Symbol;Acc:HGNC:2735] | 11029 | -0.434 | -0.2959 | Yes |
| 265 | RRS1 | ribosome biogenesis regulator 1 homolog [Source:HGNC Symbol;Acc:HGNC:17083] | 11045 | -0.437 | -0.2935 | Yes |
| 266 | DDX21 | DExD-box helicase 21 [Source:HGNC Symbol;Acc:HGNC:2744] | 11120 | -0.446 | -0.2958 | Yes |
| 267 | RPL38 | ribosomal protein L38 [Source:HGNC Symbol;Acc:HGNC:10349] | 11122 | -0.446 | -0.2922 | Yes |
| 268 | RPL6 | ribosomal protein L6 [Source:HGNC Symbol;Acc:HGNC:10362] | 11129 | -0.447 | -0.2891 | Yes |
| 269 | RPL14 | ribosomal protein L14 [Source:HGNC Symbol;Acc:HGNC:10305] | 11154 | -0.451 | -0.2873 | Yes |
| 270 | MYBBP1A | MYB binding protein 1a [Source:HGNC Symbol;Acc:HGNC:7546] | 11188 | -0.456 | -0.2862 | Yes |
| 271 | RPS5 | ribosomal protein S5 [Source:HGNC Symbol;Acc:HGNC:10426] | 11206 | -0.458 | -0.2838 | Yes |
| 272 | ATR | ATR serine/threonine kinase [Source:HGNC Symbol;Acc:HGNC:882] | 11231 | -0.461 | -0.2820 | Yes |
| 273 | AGO2 | argonaute RISC catalytic component 2 [Source:HGNC Symbol;Acc:HGNC:3263] | 11246 | -0.463 | -0.2793 | Yes |
| 274 | RPL11 | ribosomal protein L11 [Source:HGNC Symbol;Acc:HGNC:10301] | 11252 | -0.464 | -0.2760 | Yes |
| 275 | PRPF19 | pre-mRNA processing factor 19 [Source:HGNC Symbol;Acc:HGNC:17896] | 11269 | -0.467 | -0.2734 | Yes |
| 276 | URB2 | URB2 ribosome biogenesis homolog [Source:HGNC Symbol;Acc:HGNC:28967] | 11291 | -0.470 | -0.2713 | Yes |
| 277 | ERI2 | ERI1 exoribonuclease family member 2 [Source:HGNC Symbol;Acc:HGNC:30541] | 11320 | -0.474 | -0.2697 | Yes |
| 278 | PAK1IP1 | PAK1 interacting protein 1 [Source:HGNC Symbol;Acc:HGNC:20882] | 11345 | -0.478 | -0.2677 | Yes |
| 279 | METTL16 | "methyltransferase 16, N6-methyladenosine [Source:HGNC Symbol;Acc:HGNC:28484]" | 11346 | -0.478 | -0.2638 | Yes |
| 280 | DDX56 | DEAD-box helicase 56 [Source:HGNC Symbol;Acc:HGNC:18193] | 11386 | -0.484 | -0.2629 | Yes |
| 281 | WDR3 | WD repeat domain 3 [Source:HGNC Symbol;Acc:HGNC:12755] | 11429 | -0.492 | -0.2623 | Yes |
| 282 | RIOX2 | ribosomal oxygenase 2 [Source:HGNC Symbol;Acc:HGNC:19441] | 11444 | -0.495 | -0.2594 | Yes |
| 283 | LAS1L | LAS1 like ribosome biogenesis factor [Source:HGNC Symbol;Acc:HGNC:25726] | 11474 | -0.498 | -0.2576 | Yes |
| 284 | DDX52 | DExD-box helicase 52 [Source:HGNC Symbol;Acc:HGNC:20038] | 11484 | -0.499 | -0.2543 | Yes |
| 285 | CELF3 | CUGBP Elav-like family member 3 [Source:HGNC Symbol;Acc:HGNC:11967] | 11510 | -0.502 | -0.2522 | Yes |
| 286 | USP36 | ubiquitin specific peptidase 36 [Source:HGNC Symbol;Acc:HGNC:20062] | 11560 | -0.508 | -0.2519 | Yes |
| 287 | TSC1 | TSC complex subunit 1 [Source:HGNC Symbol;Acc:HGNC:12362] | 11575 | -0.511 | -0.2489 | Yes |
| 288 | PWP2 | PWP2 small subunit processome component [Source:HGNC Symbol;Acc:HGNC:9711] | 11604 | -0.516 | -0.2469 | Yes |
| 289 | WDR55 | WD repeat domain 55 [Source:HGNC Symbol;Acc:HGNC:25971] | 11605 | -0.517 | -0.2427 | Yes |
| 290 | NUFIP1 | nuclear FMR1 interacting protein 1 [Source:HGNC Symbol;Acc:HGNC:8057] | 11614 | -0.519 | -0.2391 | Yes |
| 291 | LUC7L | LUC7 like [Source:HGNC Symbol;Acc:HGNC:6723] | 11627 | -0.521 | -0.2358 | Yes |
| 292 | UTP20 | UTP20 small subunit processome component [Source:HGNC Symbol;Acc:HGNC:17897] | 11656 | -0.526 | -0.2338 | Yes |
| 293 | RPS6 | ribosomal protein S6 [Source:HGNC Symbol;Acc:HGNC:10429] | 11662 | -0.526 | -0.2299 | Yes |
| 294 | SF1 | splicing factor 1 [Source:HGNC Symbol;Acc:HGNC:12950] | 11664 | -0.527 | -0.2257 | Yes |
| 295 | RPS15 | ribosomal protein S15 [Source:HGNC Symbol;Acc:HGNC:10388] | 11671 | -0.527 | -0.2219 | Yes |
| 296 | WDR46 | WD repeat domain 46 [Source:HGNC Symbol;Acc:HGNC:13923] | 11683 | -0.529 | -0.2184 | Yes |
| 297 | DDX17 | DEAD-box helicase 17 [Source:HGNC Symbol;Acc:HGNC:2740] | 11716 | -0.535 | -0.2166 | Yes |
| 298 | RPS16 | ribosomal protein S16 [Source:HGNC Symbol;Acc:HGNC:10396] | 11755 | -0.541 | -0.2152 | Yes |
| 299 | RRN3 | "RRN3 homolog, RNA polymerase I transcription factor [Source:HGNC Symbol;Acc:HGNC:30346]" | 11762 | -0.543 | -0.2113 | Yes |
| 300 | RPL27 | ribosomal protein L27 [Source:HGNC Symbol;Acc:HGNC:10328] | 11765 | -0.544 | -0.2070 | Yes |
| 301 | LSG1 | large 60S subunit nuclear export GTPase 1 [Source:HGNC Symbol;Acc:HGNC:25652] | 11783 | -0.546 | -0.2039 | Yes |
| 302 | PTEN | phosphatase and tensin homolog [Source:HGNC Symbol;Acc:HGNC:9588] | 11836 | -0.556 | -0.2035 | Yes |
| 303 | PDCD11 | programmed cell death 11 [Source:HGNC Symbol;Acc:HGNC:13408] | 11847 | -0.559 | -0.1998 | Yes |
| 304 | PRPF39 | pre-mRNA processing factor 39 [Source:HGNC Symbol;Acc:HGNC:20314] | 11854 | -0.560 | -0.1957 | Yes |
| 305 | GTPBP4 | GTP binding protein 4 [Source:HGNC Symbol;Acc:HGNC:21535] | 11861 | -0.562 | -0.1916 | Yes |
| 306 | GAR1 | GAR1 ribonucleoprotein [Source:HGNC Symbol;Acc:HGNC:14264] | 11883 | -0.568 | -0.1886 | Yes |
| 307 | SRPK1 | SRSF protein kinase 1 [Source:HGNC Symbol;Acc:HGNC:11305] | 11889 | -0.569 | -0.1844 | Yes |
| 308 | HEATR3 | HEAT repeat containing 3 [Source:HGNC Symbol;Acc:HGNC:26087] | 11901 | -0.572 | -0.1806 | Yes |
| 309 | NOP56 | NOP56 ribonucleoprotein [Source:HGNC Symbol;Acc:HGNC:15911] | 11939 | -0.580 | -0.1788 | Yes |
| 310 | DDX54 | DEAD-box helicase 54 [Source:HGNC Symbol;Acc:HGNC:20084] | 11969 | -0.587 | -0.1764 | Yes |
| 311 | MDN1 | midasin AAA ATPase 1 [Source:HGNC Symbol;Acc:HGNC:18302] | 11973 | -0.588 | -0.1718 | Yes |
| 312 | RRP1 | ribosomal RNA processing 1 [Source:HGNC Symbol;Acc:HGNC:18785] | 11996 | -0.592 | -0.1687 | Yes |
| 313 | PPAN | peter pan homolog [Source:HGNC Symbol;Acc:HGNC:9227] | 12022 | -0.597 | -0.1659 | Yes |
| 314 | RPS27 | ribosomal protein S27 [Source:HGNC Symbol;Acc:HGNC:10416] | 12071 | -0.612 | -0.1647 | Yes |
| 315 | KRI1 | KRI1 homolog [Source:HGNC Symbol;Acc:HGNC:25769] | 12074 | -0.613 | -0.1599 | Yes |
| 316 | EXOSC2 | exosome component 2 [Source:HGNC Symbol;Acc:HGNC:17097] | 12100 | -0.620 | -0.1568 | Yes |
| 317 | SMN1 | "survival of motor neuron 1, telomeric [Source:HGNC Symbol;Acc:HGNC:11117]" | 12102 | -0.621 | -0.1518 | Yes |
| 318 | VCX | variable charge X-linked [Source:HGNC Symbol;Acc:HGNC:12667] | 12110 | -0.623 | -0.1473 | Yes |
| 319 | RPS17 | ribosomal protein S17 [Source:HGNC Symbol;Acc:HGNC:10397] | 12130 | -0.626 | -0.1437 | Yes |
| 320 | SART3 | "spliceosome associated factor 3, U4/U6 recycling protein [Source:HGNC Symbol;Acc:HGNC:16860]" | 12161 | -0.633 | -0.1410 | Yes |
| 321 | RPL13A | ribosomal protein L13a [Source:HGNC Symbol;Acc:HGNC:10304] | 12200 | -0.645 | -0.1387 | Yes |
| 322 | PTBP2 | polypyrimidine tract binding protein 2 [Source:HGNC Symbol;Acc:HGNC:17662] | 12211 | -0.649 | -0.1342 | Yes |
| 323 | DICER1 | "dicer 1, ribonuclease III [Source:HGNC Symbol;Acc:HGNC:17098]" | 12215 | -0.650 | -0.1292 | Yes |
| 324 | RPS24 | ribosomal protein S24 [Source:HGNC Symbol;Acc:HGNC:10411] | 12223 | -0.652 | -0.1244 | Yes |
| 325 | CPSF7 | cleavage and polyadenylation specific factor 7 [Source:HGNC Symbol;Acc:HGNC:30098] | 12251 | -0.661 | -0.1212 | Yes |
| 326 | NOC2L | NOC2 like nucleolar associated transcriptional repressor [Source:HGNC Symbol;Acc:HGNC:24517] | 12258 | -0.663 | -0.1163 | Yes |
| 327 | RPLP0 | ribosomal protein lateral stalk subunit P0 [Source:HGNC Symbol;Acc:HGNC:10371] | 12285 | -0.672 | -0.1129 | Yes |
| 328 | DDX31 | DEAD-box helicase 31 [Source:HGNC Symbol;Acc:HGNC:16715] | 12288 | -0.672 | -0.1076 | Yes |
| 329 | RPL3 | ribosomal protein L3 [Source:HGNC Symbol;Acc:HGNC:10332] | 12350 | -0.691 | -0.1068 | Yes |
| 330 | DDX27 | DEAD-box helicase 27 [Source:HGNC Symbol;Acc:HGNC:15837] | 12359 | -0.695 | -0.1018 | Yes |
| 331 | NOP2 | NOP2 nucleolar protein [Source:HGNC Symbol;Acc:HGNC:7867] | 12372 | -0.699 | -0.0970 | Yes |
| 332 | CELF1 | CUGBP Elav-like family member 1 [Source:HGNC Symbol;Acc:HGNC:2549] | 12391 | -0.704 | -0.0927 | Yes |
| 333 | EIF3A | eukaryotic translation initiation factor 3 subunit A [Source:HGNC Symbol;Acc:HGNC:3271] | 12402 | -0.707 | -0.0878 | Yes |
| 334 | BMS1 | BMS1 ribosome biogenesis factor [Source:HGNC Symbol;Acc:HGNC:23505] | 12403 | -0.707 | -0.0820 | Yes |
| 335 | WDR43 | WD repeat domain 43 [Source:HGNC Symbol;Acc:HGNC:28945] | 12424 | -0.716 | -0.0778 | Yes |
| 336 | RPL7 | ribosomal protein L7 [Source:HGNC Symbol;Acc:HGNC:10363] | 12519 | -0.757 | -0.0791 | Yes |
| 337 | RPS28 | ribosomal protein S28 [Source:HGNC Symbol;Acc:HGNC:10418] | 12526 | -0.761 | -0.0734 | Yes |
| 338 | GLUL | glutamate-ammonia ligase [Source:HGNC Symbol;Acc:HGNC:4341] | 12527 | -0.762 | -0.0672 | Yes |
| 339 | NPM1 | nucleophosmin 1 [Source:HGNC Symbol;Acc:HGNC:7910] | 12530 | -0.762 | -0.0611 | Yes |
| 340 | BOP1 | BOP1 ribosomal biogenesis factor [Source:HGNC Symbol;Acc:HGNC:15519] | 12580 | -0.785 | -0.0586 | Yes |
| 341 | CHD7 | chromodomain helicase DNA binding protein 7 [Source:HGNC Symbol;Acc:HGNC:20626] | 12591 | -0.791 | -0.0530 | Yes |
| 342 | YTHDF2 | YTH N6-methyladenosine RNA binding protein 2 [Source:HGNC Symbol;Acc:HGNC:31675] | 12694 | -0.860 | -0.0541 | Yes |
| 343 | AGO3 | argonaute RISC catalytic component 3 [Source:HGNC Symbol;Acc:HGNC:18421] | 12726 | -0.888 | -0.0494 | Yes |
| 344 | HSP90AA1 | heat shock protein 90 alpha family class A member 1 [Source:HGNC Symbol;Acc:HGNC:5253] | 12730 | -0.893 | -0.0423 | Yes |
| 345 | SFSWAP | splicing factor SWAP [Source:HGNC Symbol;Acc:HGNC:10790] | 12747 | -0.908 | -0.0362 | Yes |
| 346 | DDX39B | DExD-box helicase 39B [Source:HGNC Symbol;Acc:HGNC:13917] | 12783 | -0.941 | -0.0313 | Yes |
| 347 | RPL23A | ribosomal protein L23a [Source:HGNC Symbol;Acc:HGNC:10317] | 12785 | -0.944 | -0.0237 | Yes |
| 348 | DDX3X | DEAD-box helicase 3 X-linked [Source:HGNC Symbol;Acc:HGNC:2745] | 12788 | -0.949 | -0.0161 | Yes |
| 349 | TSR1 | TSR1 ribosome maturation factor [Source:HGNC Symbol;Acc:HGNC:25542] | 12814 | -0.999 | -0.0100 | Yes |
| 350 | ESF1 | ESF1 nucleolar pre-rRNA processing protein homolog [Source:HGNC Symbol;Acc:HGNC:15898] | 12821 | -1.008 | -0.0023 | Yes |
| 351 | PRPF3 | pre-mRNA processing factor 3 [Source:HGNC Symbol;Acc:HGNC:17348] | 12837 | -1.040 | 0.0050 | Yes |
Table: GSEA details [plain text format]

  

Fig 2: GOBP\_RIBONUCLEOPROTEIN\_COMPLEX\_BIOGENESIS      
 Blue-Pink O' Gram in the Space of the Analyzed GeneSet

  

Fig 3: GOBP\_RIBONUCLEOPROTEIN\_COMPLEX\_BIOGENESIS: Random ES distribution      
 Gene set null distribution of ES for **GOBP\_RIBONUCLEOPROTEIN\_COMPLEX\_BIOGENESIS**
